# Features and outcomes of unplanned hospital admissions of older people due to ill-defined (R-coded) conditions: Retrospective analysis of hospital admissions data in England

**DOI:** 10.1186/1471-2318-11-62

**Published:** 2011-10-18

**Authors:** Bronagh Walsh, Helen C Roberts, Peter G Nicholls

**Affiliations:** 1 Faculty of Health Sciences, University of Southampton, Highfield, Southampton, UK; 2 Academic Geriatric Medicine, University of Southampton, Southampton General Hospital, Tremona Road, Southampton, UK

## Abstract

**Background:**

Rising rates of unplanned admissions among older people are placing unprecedented demand on health services internationally. Unplanned hospital admissions for ill-defined conditions (coded with an R prefix within Chapter XVIII of the International Classification of Diseases-10) have been targeted for admission avoidance strategies, but little is known about these admissions. The aim of this study was to determine the incidence and factors predicting ill-defined (R-coded) hospital admissions of older people and their association with health outcomes.

**Methods:**

Retrospective analysis of unplanned hospital admissions to general internal and geriatric medicine wards in one hospital over 12 months (2002) with follow-up for 36 months. The study was carried out in an acute teaching hospital in England. The participants were all people aged 65 and over with unplanned hospital admissions to general internal and geriatric medicine. Independent variables included time of admission, residence at admission, route of admission to hospital, age, gender, comorbidity measured by count of diagnoses. Main outcome measures were primary diagnosis (ill-defined versus other diagnostic code), death during the hospital stay, deaths to 36 months, readmissions within 36 months, discharge destination and length of hospital stay.

**Results:**

Incidence of R-codes at discharge was 21.6%, but was higher in general internal than geriatric medicine (25.6% v 14.1% respectively). Age, gender and co-morbidity were not significant predictors of R-code diagnoses. Admission via the emergency department (ED), out of normal general practitioner (GP) hours, under the care of general medicine and from non-residential care settings increased the risk of receiving R-codes. R-coded patients had a significantly shorter length of stay (5.91 days difference, 95% CI 4.47, 7.35), were less likely to die (hazard ratio 0.71, 95%CI 0.59, 0.85) at any point, but were as likely to be readmitted as other patients (hazard ratio 0.96 (95% CI 0.88, 1.05).

**Conclusions:**

R-coded diagnoses accounted for 1/5 of emergency admission episodes, higher than anticipated from total English hospital admissions, but comparable with rates reported in similar settings in other countries. Unexpectedly, age did not predict R-coded diagnosis at discharge. Lower mortality and length of stay support the view that these are avoidable admissions, but readmission rates particularly for further R-coded admissions indicate on-going health care needs. Patient characteristics did not predict R-coding, but organisational features, particularly admission via the ED, out of normal GP hours and via general internal medicine, were important and may offer opportunity for admission reduction strategies.

## Background

Rising demand for healthcare within ageing populations is an international phenomenon and presents challenges for efficient delivery of healthcare in many countries. In the UK, the recent increases observed in unplanned hospital admissions of older people have been described as unsustainable for the National Health Service (NHS) [1,2]. Policy has therefore focused on admission avoidance interventions such as community case management for high risk patient groups, but this approach has had limited success in the UK and elsewhere [3,4]. Improved targeting of avoidable admissions has therefore become a priority.

In the UK, older people's admissions to hospital for ill-defined conditions have been identified as likely to be avoidable [2,5,6]. In England and Wales, all inpatient admission episodes in the NHS are recorded by hospitals. The data are collated in a national database [7], which includes information on primary and secondary disease codes contributing to the inpatient stay; similar systems are used in other countries. Internationally, diagnoses are coded using the World Health Organisation (WHO) International Classification of Diseases (ICD) [8], version 9 or 10. Admissions for ill-defined conditions (R-codes) are coded with an R prefix within Chapter XVIII of the ICD-10 ('Symptoms, signs and abnormal laboratory findings'), equivalent to codes 7800 to 7990 in Chapter XIV ('Symptoms, signs and ill-defined conditions') of the ICD-9, from which the term ill-defined conditions has remained in use. Unplanned hospital admissions for ill-defined conditions are known to be increasing in the UK and other countries and they are a common feature of older people's admissions, demonstrating a stepwise increase in incidence with age even within older populations [9-15]. In older people, national hospital admissions statistics report incidence varying from approximately 7% in the US and UK [7,15] to 9% in Australia [16]. Ill-defined conditions admissions have been highlighted as a target for admission reduction strategies under the assumption that they are a consequence of increased prevalence of inadequately managed chronic disease in the ageing population and because they may be avoidable through improved chronic disease management in the community [6,17]. It has also been suggested that these admissions represent inappropriate acute hospitalisations from nursing homes or in older people at the end of life. However, analysis of national data suggests that health service organisational factors, such as access to alternative services and changes to admission procedures may be more important than ageing and chronic disease [11,18].

Improved understanding of the relationship between demographic, patient and organisational factors and incidence and outcomes of these admissions could improve the targeting of admission avoidance interventions with beneficial consequences for health services and patients. Such information would be relevant both to health services with high rates of acute admissions for ill-defined conditions and to areas (for example the ED, acute medicine and nursing homes) where they are commonly encountered [11,14,19]. In this case, the focus for investigation was ill-defined hospital admissions in acute inpatient medical settings where managing demand for unplanned admissions is a priority and where there was little information about the incidence and outcomes of these admissions. The objectives of this study were therefore to determine: the incidence of R-codes at discharge from a combined general internal and geriatric medicine directorate; the admission and patient characteristics R-coded episodes, particularly those which might indicate potential drivers of admission such as comorbidity and referral from nursing home; factors predicting an R-coding at discharge; and outcomes (mortality, length of stay and discharge destination) for R-coded and other patients.

## Methods

The study comprised a retrospective analysis of unplanned hospital admissions. Data were extracted from the Patient Administration System (PAS) of an acute NHS Hospital Trust in England on all unplanned admissions of people aged ≥65 to general internal and geriatric medicine during 2002. Subsequently, deaths and readmissions at 36 months were extracted for individuals identified in the first data set. The hospital in which data were collected is a large (1100 bed), teaching hospital in the south of England. It serves a mixed urban and rural population of approximately 1.3 million people. The population is largely White British, with approximately 8% non-white population, mostly South Asian in origin. Overall mortality rates, deprivation levels, health indicators and age structure of the population reflect the pattern for England as a whole [20]. A data-sampling period of 12 months ensured that seasonal effects were avoided and resulted in a sample size in excess of 5000. A sample size of the order 4300 gives a margin of error of +/- 1.5% around the incidence estimates. Data were extracted retrospectively in 2006-2007 to ensure completeness of the data set including 36-month follow-up. Data included: gender; age; residence (community dwelling or residential care); route (admission to inpatient ward from the ED or directly via the GP); time of admission (during standard GP hours or out of hours; in the NHS out of hours provision is between 18.30 and 08.00 and during all weekends and public holidays); comorbidity measured by a count of total diagnostic codes for the admission; discharge destination; deaths in hospital and up to 36 months after discharge; re-admissions up to 36 months after discharge; number of within-hospital transfers (patients may transfer from admission or observation units to inpatient wards or between inpatient wards and lack of continuity in care may contribute to ill-defined diagnoses); primary discharge diagnostic code (using ICD-10) at first and subsequent admissions; medical speciality at discharge (general internal or geriatric medicine; usually patients aged 75 and over are admitted to the latter); and length of hospital stay. Cases with a primary code of R69X (unknown and unspecified causes of morbidity) and R95-99 (ill-defined and unknown causes of mortality) were to be excluded from the analyses since these episodes are un-coded or not yet coded and are thus effectively missing data. Approval for this study was obtained from the Southampton and South West Hampshire Research Ethics Committee (study number 04/Q/1704/17).

## Analysis

Data were analysed using SPSS release 16.0 and STATA 9.0. The first stage of the analysis focused on features of the admission episode. Descriptive statistics were calculated for the whole sample and for sub-groups. Chi- squared tests were used to compare groups on diagnosis, discharge destination, gender and time of admission. Kruskal-Wallis equality of populations rank tests were used for comparisons of age, numbers of within-hospital transfers and length of stay due to the skewed distributions of these variables. Logistic regression was used to investigate the relative contribution of age, gender, number of diagnoses, residence, time of admission and speciality to diagnostic category at discharge for these admissions. In the second stage of the analysis, follow-up data were extracted on the individuals identified from the initial dataset. Chi- squared tests were used to compare groups on mortality and readmissions. Hazard ratios were used to explore the effect of diagnostic category at first admission on mortality and readmission in the longitudinal follow-up data.

## Results

Data were extracted for 6760 admissions during 2002, representing 5386 individuals, with follow-up data available for 5312 individuals after removal of incomplete and anomalous records. There were no exclusions for diagnoses (R69X or R95-99) as no individuals received these codes at the first admission. Mean age was 80.9 years (range 65-104). The admissions included 2898 (43%) males and 3862 (57%) females. The admission source was the patient's usual residence in the community in 6665 (98.6%) of cases, with only 54 (0.8%) identified as admitted from residential care. At discharge, 1461 admissions (21.6%) were allocated R-codes, whilst 5299 (78.4%) were allocated other diagnoses. The five most common symptom diagnoses within the R-coded group were circulatory (including chest pain, syncope and collapse) (28%), respiratory (28%), senility (14%), abdominal pain (11%) and cognitive symptoms (6%), which accounted for 87% of the R-coded admissions. Within the other group, the main five diagnostic categories were cardiovascular (32%), respiratory (21%), gastrointestinal (12%), cancers and blood disorders (8%) and injuries and accidents (7%), accounting for 80% of the other admissions.

Analysis of the characteristics of the R-coded and other admissions (Table 1) indicates that R-coded admissions were significantly more likely to be admitted directly from the ED whereas other admissions were more likely to have been admitted via their GP, and were significantly more likely to have entered hospital during GP out-of-hours service times. They were significantly less likely to be admitted from residential care. R-coded admissions were also significantly less likely in geriatric medicine. The R-coded admissions had a substantially shorter length of stay, lower numbers of within-hospital transfers, and were more likely to be discharged to their usual residence. Age at admission was slightly, but significantly, younger for the R-coded than the other admissions. Fewer of the R-coded admissions ended in death during the period of hospitalisation. No differences were observed in gender or number of disease diagnoses.

**Table 1:** Admission features and discharge outcomes for R-coded and other admissions

Number (%)	Other codes n = 5299 (78.4)	R-codes n = 1461 (21.6)	P-value (X^2^)
**Gender**			
Male	2275 (42.9)	623 (42.6)	0.43
Female	3024 (57.1)	838 (57.4)	
			
**Source of admission**			
Usual residence	5214 (98.4)	1451 (99.3)	0.03
Residential care	5 (0.1)	0	
Other	80 (1.5)	10 (0.07)	
**Route of admission**			
ED	2421 (45.7)	897 (61.4)	<0.001
GP	2578 (48.7)	515 (35.2)	
Other	300 (5.7)	49 (3.4)	
**Time of admission**			
Out of hours	2332 (44.0)	749 (51.3)	<0.001
Normal GP hours	2967 (56.0)	712 (48.7)	
**Speciality**			
Internal Medicine	3307 (62.4)	1135 (77.7)	<0.001
Geriatric Medicine	1992 (36.6)	326 (22.3)	
**Discharge destination**			
Usual residence	3558 (67.1)	1231 (84.3)	<0.001
Residential care	121 (2.3)	19 (1.3)	
Other hospital	467 (8.8)	104 (7.1)	
Other	192 (3.6)	37 (2.5)	
Deaths In hospital	961 (18.1)	70 (4.8)	

**Median (IQR)**			**P-value** **(Kruskal-Wallis)**

**Age**	81 (12)	79 (12)	<0.001
**No. diagnoses**	1 (1)	1 (1)	0.57
**No. transfers**	1 (1)	1 (2)	<0.001
**Length of stay**	8 (16)	4 (9)	<0.001

Logistic regression analysis (including age, gender, residence, speciality, route of admission and time of admission) indicated that odds of receiving an R-code at discharge were decreased by admission to geriatric rather than general internal medicine (odds ratio (95%CI) 0.51 (0.43, 0.59) p < 0.001), admission from residential care (odds ratio (95%CI) 0.61 (0.45, 0.82) p < 0.001), and admission via GP rather than the ED (odds ratio (95%CI) 0.61 (0.54, 0.69) p < 0.001). Age, gender, time of admission and number of medical conditions were not predictive of R-coding. Predictors of R coded discharges were also explored using Cox proportional hazard regression analysis, allowing adjustment for multiple admissions by the same individual. However, this alternative approach to the analysis confirmed the findings of the logistic regression presented here. The analysis was repeated for elderly care admissions alone where only admission via the ED remained a significant predictor of R-code at discharge (odds ratio (95%CI) 0.57 (0.45, 0.73) p < 0.001).

Chi-squared and Cox proportional hazard analyses were carried out on the 5312 individuals identified from the first data extraction exercise classified according to diagnostic category at discharge from the first admission (Table 2). Significantly more R-coded patients survived the follow-up period. No significant differences were observed in readmission rates. Mortality up to 36 months from first admission demonstrated that the hazard ratio for R-coded compared with other patients was 0.71 (95%CI 0.59, 0.85 p < 0.001). Readmission was analysed for the 4548 individuals surviving at first discharge from hospital. For readmission the hazard ratio was 0.96 (95% CI 0.88, 1.05 p = 0.487). However, hazard ratio for survival to a further R-coded readmission episode for R-coded compared with other patients was 1.57 (95%CI 1.34, 1.83 p < 0.001) indicating that the risk of readmission for ill-defined conditions was significantly higher in those originally admitted with these problems.

**Table 2:** Number (%) of deaths and readmissions up to 36 months for R and other patients

	Other codes n = 4165 (78.4)	R-codes n = 1147 (21.6)	P-value (X^2^)
**Death**			
In hospital	716 (17.2)	48 (4.2)	<0.001
Within one month	64 (1.5)	10 (0.9)	
1 to12 months	356 (8.5)	79 (6.9)	
13 to 36 months	218 (5.2)	59 (5.1)	
Survived	2811 (67.5)	951 (82.9)	

	**n = 3449**	**n = 1099**	

**Readmission^1^**			
Within 30 days	336 (9.7)	115 (10.5)	0.50
Within one year	1115 (32.3)	333 (30.3)	
Not readmitted	1998 (57.9)	651 (59.2)	

^1 ^Readmissions calculated for the 4548 out of 5312 individuals surviving at first discharge

## Discussion

This is the first study to focus specifically on describing the incidence and characteristics of unplanned internal medical and geriatric inpatient admissions for ill-defined (R-coded) conditions amongst older people. This study uses routine data that is collected in all NHS hospitals in England which, combined with the typical demographic profile of the local population, means that relevance and generalisability to other UK settings is high. In addition, the ICD coding system is used internationally to describe patterns of disease and mortality and is a feature of health services datasets in many countries, enhancing transferability and enabling international comparisons to be made. It should be noted that an important limitation of this study is that this retrospective analysis was necessarily limited by being confined to routinely available information. It can therefore only be viewed as a preliminary investigation into these admissions. However, this study has provided some useful indicators that this type of admission may be worthy of further research.

Nearly 22% of the sample received R-codes, suggesting that the incidence rate in acute medical settings is higher than would be predicted from international data on all hospital admissions amongst older people. This finding contrasts with estimates of up to 10% of all hospital admissions internationally [21] and 7% in England [11], but is consistent with higher rates in studies focused on urgent care settings [9,10,14]. If these are indeed avoidable admissions, the scale of the problem presented by ill-defined conditions in acute medical settings may have been underestimated. The high incidence in older people could be viewed as unsurprising given that non-specific illness presentation is known to be a characteristic of the older patient. The ICD system lacks discrimination in older patients; geriatric syndromes such as recurrent falls are not easily classified and may well be allocated R-codes [10,11,22,23]. However, in this study, R-codes were less common in geriatric medicine (14.1%) than in general internal medicine (25.6%). Age was not a predictor of R-coded admission diagnoses in this patient group. Co-morbidity, measured by mean number of diagnoses, was equally common in the R-coded patients as those with other diagnostic codes. This does not preclude chronic disease being the underlying cause of the admissions, but does make identification of those at risk problematic and does not indicate that improved chronic disease management would have a specific effect on these admissions. It should however be noted that co-morbidity was measured in this study by a simple count of the number of diagnoses at discharge, a common method of assessing overall chronic disease burden [24]. However, it is known that discharge summaries (on which disease coding is based) tend to under-represent secondary and underlying conditions. Further investigation of co-morbidity in a prospective study using a more robust method of measurement would be useful in the future, but was not possible in this study because of insufficient information within the routine dataset.

When interpreting these findings it is important to note that coding is notoriously problematic, with varying estimates of error rates. In the UK, specially trained coding teams are employed by hospitals to allocate disease codes based on the discharge summary written by the medical team. Errors may result from the discharge summary being completed by less experienced physicians [25]. It is also known that coding errors are more likely for rare conditions, whereas common cardiovascular and respiratory illnesses have accuracy of approximately 97% [26,27]. It has also been shown that coding accuracy improves at approximately 4-11% per year in the first few years following introduction of a new system [28]. In this study, the ICD-10 had been in use since 1999 and the majority of diagnostic codes are commonly used, suggesting that coding accuracy should have been high. In addition, the level of coding used in this analysis (chapter rather than specific disease level) is known to be more accurate (at 81-92%) than more detailed codes [29,30].

The observed difference in incidence of these admissions between general internal medicine and geriatric medicine, and the importance of speciality as a predictor of the R-code, also raises a question about the allocation of codes within the two specialities. Since this study was carried out within a combined directorate with one coding team it is unlikely that different coding practices apply, although different patterns of reporting the discharge summary might occur between general and geriatric medical teams. It is therefore possible that the lower incidence in geriatric medicine resulted from a higher likelihood of receiving other diagnostic codes, perhaps due to a greater emphasis on recording underlying chronic conditions, or from a lower likelihood of being admitted by these teams. Further research would be needed to determine which is the case, but sub-group analyses (*supplementary results available *) suggest that the geriatric medicine patients had more acute problems rather than them being less likely to be R-coded. It is also possible that coding is influenced by external factors such as differentials in reimbursement rates for specific conditions or procedures. However, in this case the current system of payment to hospitals in England attracts a lower tariff for R-coded conditions, so there should be no incentive to use this set of codes. Despite this, R-coded admissions have continued to rise in recent years.

The question remains as to whether R-codes are a real clinical phenomenon or a consequence of missed or incorrect diagnoses. In the latter case, length of stay may be important. A short length of stay, as observed in this study for the R-coded patients, might result in less opportunity for investigation and diagnosis, leading to a less informative discharge summary from the physician and less likelihood of a defined cause for admission at coding. This might be more likely in the case of older people with multiple morbidity and atypical disease presentation. However, it is also the case that shorter length of stay may genuinely reflect clinical need and the resultant decision to discharge the patient more rapidly. In this study, the differences in outcomes between the two diagnostic groups appear to support the suggestion of less serious illness in the R-coded group. However, the mortality rate in the R-code group, whilst significantly lower than that for other diagnoses, was still substantial. This suggests the need for careful analysis of patient needs and systems to ensure appropriate assessment is in place if this group are to become a focus for admission avoidance interventions. Priorities for the future will be to describe the patient group in more detail via prospective studies focusing on the impact of the type and severity of comorbidity, functional and cognitive deficits that are not available in the routine datasets. Assumptions within current UK health policy that rapid rises in R-coded admissions are a consequence of poorly managed chronic disease and an ageing population require further investigation.

The lower incidence in those admitted via the GP, during standard GP hours and to geriatric medicine suggests that health service organisational factors may be more important in rising admissions for ill-defined conditions than chronic disease and ageing *per se *; in England, as in other developed countries, recent decades have seen declines in availability of acute hospital beds and moves towards community-based care, but these have occurred against a backdrop of reorganisation of out of hours care that has reduced access to a known GP. The regression models indicated that admission route was the only significant predictor of R-coding once medical speciality was taken into account. The potential influence of changes to practice such as out-of-hours provision and the introduction of financial penalties for exceeding the four hour waiting time limit in the ED has been highlighted elsewhere [31], but could be a driver of the higher admission rates via the ED and out-of-hours. Overall, these data support the premise that these organisational factors are a major driver for R-codes in England [11]. Given this, specialist geriatric assessment and access to alternative services through primary care could be key factors in controlling admissions of this type, but identifying these patients for specialist input during admission could be difficult since patient features were not predictive of receiving an R-code. One avenue would be to follow up patients who are R-coded at discharge to prevent similar subsequent admissions for ill-defined admissions, which appear from these data to be more likely for the R-coded group. Prospective studies would allow more information to be gathered on functional and social support prior to admission and access to alternative services at the point of hospitalisation. International comparisons of incidence data could also be revealing if viewed in the context of different admissions systems and health services.

## Conclusions

The high number of unplanned R-coded admissions occurring within the context of rapidly rising admissions amongst older people has attracted policy attention internationally focussed on admission reduction strategies. It has been assumed that these admissions are the result of poor chronic disease management and are avoidable with appropriate preventative and community care. In this study the factors predicting R-coding were organisational in nature including admission route and time, while the shorter length of stay, lower mortality, and lower proportion discharged to residential care in the R-coded group supports the contention that such admissions may be in less need of acute care and may be avoidable if appropriate services are in place. However, the high rate of readmission, particularly for further R-coded conditions in those initially admitted for ill-defined conditions, indicates that these patients have on-going health care needs requiring further investigation and management. Interestingly, although previous national and international analyses of older people's admissions indicated that these admissions increase with age, in this study the patients admitted via elderly care were less likely to be R-coded. There may therefore be scope for applying best practice from elderly care more widely to reduce such admissions in future.

## Competing interests

All authors want to declare: (1) no financial support for the submitted work from anyone other than their employer. All authors also declare: (2) No financial relationships with commercial entities that might have an interest in the submitted work; (3) No spouses, partners, or children with relationships with commercial entities that might have an interest in the submitted work; and (4) No non-financial interests that may be relevant to the submitted work.

## Authors' contributions

BW and HCR were involved in study concept and design, data analysis and interpretation. BW was responsible for data extraction, database management, data analysis and study coordination. PGN was responsible for database management, data analysis and data interpretation. All authors were involved in preparation of the manuscript and all authors have read and approved the final manuscript. BW is the guarantor.

## Pre-publication history

The pre-publication history for this paper can be accessed here:

http://www.biomedcentral.com/1471-2318/11/62/prepub
